# Septic knee arthritis in Crohn’s disease biological therapy-free patient. Case report

**Published:** 2015

**Authors:** C Pop, D Calagiu, P Jantea, R Nemes

**Affiliations:** * ”Carol Davila” University of Medicine and Pharmacy, Bucharest, Romania; ** Medical Clinic and Gastroenterology, University Emergency Hospital, Bucharest, Romania; *** ”Marius Nasta” National Institute of Pneumology, Bucharest, Romania

**Keywords:** Crohn’s disease, septic arthritis, synovial fluid, phlegmonous mass

## Abstract

A 52-year-old woman with Crohn’s disease presented with septic arthrtis of the knee. This condition coincided with a symptomatic flare of her Crohn’s disease due to an ileal inflammatory stenosis, manifested as a phlegmonous mass palpable in the right lower quadrant and a small bowel obstruction. Results of synovial fluid cultures showed the presence of Gram-negative bacillus, Klebsiella pneumoniae and the CT scan images were highly suggestive of abdominal abscess within Crohn’s disease. The patient’s condition improved after following an antibiotic treatment and after the initiation of Anti-TNF-alpha agent Adalimumab, with no further exacerbation. Septic arthritis in Crohn’s disease should be considered to have a communicating source of sepsis consisting of an abdominal abscess or fistula.

**Abbreviations:** Anti-TNF-alpha agent = anti tumor necrosis factor alpha agent, 5-ASA = 5-aminosalicylic acid

## Introduction

Crohn’s disease is a chronic inflammatory bowel condition of unknown etiology affecting any part of the gastrointestinal tract, with intermittent activity throughout the patient’s life [**2**]. The transmural nature of the disease determines three distinct events: inflamatory, fistulizing and stenosing. Stricture is a characteristic complication of Crohn’s disease and represents a long-standing inflammation occuring in the segment in which the inflammmation has been active [**2**]. Numerous extraintestinal manifestations may also be present. Among the most common extraintestinal manifestations there are disorders of the joints - arthritis of knee, ankle and any of meta-carpo/ tarsophalangeal joints [**1**,**15**]. Glucorticoids used to treat this affection can lead to an aseptic necrosis of the hip [**7**,**8**]. A septic arthritis, although a rare complication of Crohn’s disease, should be kept in mind.

We present a case of septic arthritis of the knee related to a flare up of ileal Crohn’s disease due to an ileal inflammatory stenosis.

## Case report

A 52-year-old woman with a 7-year history of Crohn’s disease controlled by 5-ASA derivated and Azathioprine, presented with a short history of a swollen right knee accompanied by significant pain and functional impotence, fever and chills. Symptoms began suddenly, with abdominal complaints and an episode of presyncope, followed several days after by joint manifestations.

Her past medical history was significant for two episodes of knee septic arthritis, the first, 15 years before (preceding the diagnosis of Crohn’s disease) and a second episode, 5 years before, confirmed as staphylococcal septic arthritis.

She had numerous considerable complications of several years, of long-term corticotherapy (cortico-dependent): aseptic necrosis of the hip, central obesity, impaired glucose tolerance, arterial hypertension, ischemic heart disease, and osteoporosis. 

Her medication included mesalamine, azathioprine, metoprolol, calcium, and multivitamins. 

The patient had never taken Anti-TNF-alpha agents and had been corticoid-free for two years. 

On admission, the patient had an impaired general condition, malaise, high fever of 38,50C, dehydrated skin, nausea, TAs-90mmHg, AV-110 b/ min, respiratory rate-22 b/ min, distended abdomen with lower quadrant and right flank pain to palpation, as well as extremely painful red, warm and swollen right knee accompanied by an important limitation of joint passive motion. 

Laboratory tests on admission highlighted an important inflammatory syndrome with leukocytosis-14*103 / mm3 neutrophilia 82.7%, ESR 90mm/ h CRP 299.14 U/ L and important hypokalemia 2.5 mEq/ L.

**Table 1:** Laboratory tests evolution

Laboratory test	On admission	7 days to admission	14 days to admission	Discharge	6 months later
WBC 103/ mm3	14	7.37	9.29	5.05	3.0
Hemoglobin g/ dl	13.9	11.9	11	10.8	13.3
Platelet 103/ mm3	423	452	585	372	241
Neutrophil %	82.7	62	77.8	58.7	43.3
ESR mm/ h	90	69	60	55	16
RCP	299.14	86.9	16.7	9.8	4.7
Fibrinogen mg/ dl	697.01	461.85	342.76	235.23	201.32
Na mEq/ L	149	143	139	140	138
K mEq/ L	2.5	2.9	3.9	3.5	4.1
Calcium mg/ dl	7.2	7.9	8.1	8.2	8.1
BUN mg/ dl	27	19	15	17	35
Creatinine mg/ dl	0.7	0.6	0.6	0.8	0.7
Glucose fast mg/ dl	119	110	112	108	123
Iron μg/ dl	25	18	25	29	83
Ferritin μg/ L	285	237	-	-	98
ALT U/ L	15	12	21	23	34
AST U/ L	18	20	21	24	24
Protein total g/ dl	6.21	6.11	-	5.8	6.23
Uric Acid mg/ dl	5.7	4.8	2.9	3.5	4.8
Blood cultures for aerobic and anaerobic germs	Negative	-	-	-	-

Arthrocentesis for diagnostic and therapeutic reasons was mandatory. Arthrocentesis can give a fast pain relief and increase in the range of motion, and it also allows the analysis of fluid to determine the type of arthritis [**19**-**21**]. We aspirated 80ml of yellowish, turbid synovial fluid from her right knee.

All fluid aspirated was sent for Gram stain, cell count with a leukocyte formula, aerobic and anaerobic bacteria culture. We recommended crystal analysis from the synovial fluid for differential diagnoses with crystal arthritis-gout and pseudogout. 

**Table 2:** Characteristics of synovial fluid

Synovial fluid knee joint	*On admission*	7 days to admission
Volume ml	*80*	20
Viscosity	*turbid*	turbid
Clarity	*Opaque*	Cloudy/opaque
Color	*yellowish*	Yellowish
WBC/mm3	*53.028*	38.118
Neutrophils%	*91*	82.1
Monocytes%	*17.9*	9
Gram stain	*negative*	Negative
Ziehl-Neelsen stain	*BAAR negative*	BAAR negative
Culture	*Klebsiella pneumoniae *	Negative
Crystal analysis	*negative*	-

The laboratory tests from aspirated fluid showed a number of 53,000 leukocytes/ mm3 with a predominance of neutrophils of 91%.

Based on the clinical features, risk factors and synovial fluid laboratory results, we established the diagnosis of septic arthritis and initiated an empiric antibiotic treatment. The patient was started on Imipenem and Vancomycin. Improvement of the knee was only transient, on the day following arthrocentesis she complained of knee pain and swelling of the left knee. We performed a new arthrocentesis on the contralateral knee and aspirated 30ml synovial fluid with the same aspect as the previous. Laboratory tests also showed an increased number of leucocytes 46,000/ mm3.

**Table 3:** Risk factors for septic arthritis

	Condition		**	*Present in our patient*	**
HIV infection	Skin infection	Bacteremia episodes	*no*	*no*	*May be*
Diabetes mellitus	Other chronic diseases		*Impaired glucose tolerance*	*Crohn’s disease*	**
	Immunosuppressive therapy		**	*Azathioprine*	**
Preexistent joint damage	Prosthetic joint		*Two episodes of arthritis in her medical past *	*no *	**
	Old age		**	*52 year old*	**
	Malignancy		**	*Not known*	**
	Biological therapy		**	*no*	**

Considering arthrocentesis and knee pain relief and fluid resuscitation, we recommended performing a CT scan to elucidate the cause of the tumoral palpable mass in the lower abdominal quadrant; she had abdominal pain and developed an onset of diarrheal stools; her general state, anorexia, nausea were not improved. 

Contrast-enhanced CT scan revealed luminal narrowing and mural thickening of distal ileum causing a dilatation of fluid-filled small bowel proximally and collapsed descending colon. The mural stratification of the involved segment was seen suggestive of inflammatory stenosis. There was a circumscribed ovalar fluid, water-density mass and extraluminal gas bubbles under bottom caecum, which appeared dilated. Peri intestinal fat revealed an inflammatory change. 

These changes were highly suggestive of abdominal abscess within Crohn’s disease. 

The results of synovial fluid cultures showed the presence of Gram-negative bacillus, Klebsiella pneumoniae, sensitive to Imipenem. However, two sets of blood culture were negative for any organism. We believe the septic knee was as a direct result of intra-abdominal pathology and decided to continue the antibiotic treatment with Imipenem and initiate an anti TNF-alpha agent Adalimumab for the exacerbation of Crohn’s disease. 

The patient’s condition improved following treatment, with no further exacerbation of articular symptoms, and the abdomen also settled with the medical management with Azathioprine and Adalimumab. She was discharged after three weeks in a good condition.

## Discussion

Articular manifestations in Crohn’s disease are frequent and include peripheral arthritis and axial involvement; secondary osteoporosis and hypertrophic osteoarthropathy may also occur [**3**,**4**].

Adverse effects of corticosteroid treatment, such as osteonecrosis, may also affect joints [**4**,**5**].

Septic arthritis, though rare in the case of complex Crohn’s disease, should also be taken into consideration as differential diagnosis of joint symptoms, particularly in the settings of oligoarticular arthritis. [**1**,**13**].

Bacterial arthritis is habitual due to the hematogenous seeding of a joint during a persistent or even a transient bacteremia, for example during an attack of Crohn’s colitis with abdominal abscess [**24**]. The bacteria enter the joint space and trigger an acute inflammatory synovitis. Release of cytokines and proteases leads to cartilage degradation within hours [**15**,**16**].

The diagnostic procedure of choice is an arthrocentesis. Synovial fluid is often purulent, and the leukocyte count is usually between 50,000/ mm3 and 100,000/ mm3 with > 75-90% polymorphonuclear leukocytes [**23**]. This simple laboratory test represented the cornerstone for the positive diagnosis of septic arthritis; known that another arthritide mainly gout and pseudogout can exhibit an identical clinical picture to that of septic arthritis [**5**,**6**].

Synovial fluid should be cultured for both aerobes and anaerobes; the Gram stain should be positive in 35-65% of patients. All synovial fluid should be examined for uric acid and calcium pyrophosphate crystals [**3**,**16**].

Treatment of joint symptoms in Crohn’s disease include sulphasalazine, azathioprine, methotrexate and glucocorticoids [**9**-**12**,**14**]. Anti-tumor necrosis factor antibodies are effective in treating resistant or complicated Crohn’s disease as well as peripheral arthritis [**17**,**18**].

The principles in the management of infectious arthritis include drainage of the purulent synovial fluid and the administration of appropriate parenteral antimicrobial therapy to prevent cartilage destruction. The administration should be initially empirical considering the possible bacterial etiology of a septic knee: Staphylococcus a., Enterobacteriaceae and Pseudomonas a. [**8**,**21**].

Arthroscopy is an attractive option for the initial therapy of infectious arthritis because of the improved ability to drain purulent material from the joint compared with joint aspiration [**22**,**23**].

## Conclusion

It is unexpected for a septic knee to be the most important presenting complaint in acute Crohn’s colitis. The key lesson from this case is that septic arthritis in Crohn’s disease should be considered to have a communicating source of sepsis consisting in an abdominal abscess or fistula.

**Conflicts of interest**

There are no conflicts of interest to report.

There was no funding source involved.
